# Published sequences do not support transfer of oseltamivir resistance mutations from avian to human influenza A virus strains

**DOI:** 10.1186/s12879-015-0860-9

**Published:** 2015-03-28

**Authors:** Peter Norberg, Magnus Lindh, Sigvard Olofsson

**Affiliations:** Department of Infectious Diseases and Virology, Sahlgrenska Academy, University of Göteborg, Guldhedsgatan 10 B, S-413 46 Gothenburg, Sweden

**Keywords:** Influenza A, Avian influenza, Tamiflu, Oseltamivir resistance, Resistance mutations

## Abstract

**Background:**

Tamiflu (oseltamivir phosphate ester, OE) is a widely used antiviral active against influenza A virus. Its active metabolite, oseltamivir carboxylate (OC), is chemically stable and secreted into wastewater treatment plants. OC contamination of natural habitats of waterfowl might induce OC resistance in influenza viruses persistently infecting waterfowl, and lead to transfer of OC-resistance from avian to human influenza. The aim of this study was to evaluate whether such has occurred.

**Methods:**

A genomics approach including phylogenetic analysis and probability calculations for homologous recombination was applied on altogether 19,755 neuraminidase (N1 and N2) genes from virus sampled in humans and birds, with and without resistance mutations.

**Results:**

No evidence for transfer of OE resistance mutations from avian to human N genes was obtained, and events suggesting recombination between human and avian influenza virus variants could not be traced in the sequence material studied.

**Conclusions:**

The results indicate that resistance in influenza viruses infecting humans is due to the selection pressure posed by the global OE administration in humans rather than transfer from avian influenza A virus strains carrying mutations induced by environmental exposure to OC.

**Electronic supplementary material:**

The online version of this article (doi:10.1186/s12879-015-0860-9) contains supplementary material, which is available to authorized users.

## Background

Tamiflu (oseltamivir phosphate ester, OE) is recommended by the WHO as a first line defense during influenza pandemic situations [1]. The active metabolite, oseltamivir carboxylate (OC) is secreted via urine or feces [2] and degraded only scarcely by wastewater treatment plants, which might lead to contamination of aquatic ecosystems hosting waterfowl [3]. Since influenza virus infections are persistent in waterfowl it has been postulated that presence of OC in the natural habitats of such birds could induce OC resistance among the influenza virus strains that colonize waterfowl [4,5]. This apprehension has been supported by field studies describing OC resistance mutations in influenza A virus isolated from wild birds [6], and experimentally by demonstrating rapid development of OC-resistant virus in influenza A virus-infected mallards that were kept in artificial, OC-containing environments [7]. This has raised concerns that OC-resistance mutations might be transferred to influenza A viruses that circulate among humans, thereby compromising the use of OE [8]. It is therefore important to assess, firstly, the risks for transfer of OC resistance mutations that emerge in avian influenza virus into influenza virus spreading in the human population, and, secondly, the possible influence of this phenomenon on treatment efficiency.

The influenza virus neuraminidase (N) is the molecular target for OE/OC and, hence, the viral N genes are the major carriers of resistance mutations (Reviewed in [9]). Although zoonotic transfer of avian influenza A virus to man occurs, mostly involving H5N1, H7N7, H7N2, H7N3, and H7N9 [10,11], this usually represents a dead end because further man-to-man transfer is rare. This does, however, not per se preclude the possibility that OC resistant mutations generated in avian influenza viruses could be transferred to human viruses (here the term “human influenza virus” denotes virus variants with capacity to spread in the human population) with or without involvement of swine or other animals [12] considered as “mixing vessels” for new pandemic influenza virus.

The probability of future genetic resistance transfer from avian influenza virus to viruses circulating in the human population may be assessed by evaluating past interactions and exchange of genetic material between these viruses. Thus, if transfer of resistance mutations from avian influenza virus to human influenza virus has occurred, this would have resulted in avian influenza virus sequence imprints in the N gene, some of which should appear in published human OC-resistant sequences. To check for this possibility we analyzed a large number of N1 and N2 gene sequences of human and avian influenza A viruses representing both OC-resistant and wild-type strains.

## Methods

### Avian and human influenza virus N1 and N2 genes studied

The influenza virus N1 and N2 genes discussed below were derived from specimens taken from humans (human sources) or from birds (avian sources). Altogether 10,351 N1 genes from human sources and 2,062 N1 genes from avian sources were analyzed, of which 107 genes from human sources and four genes from an avian sources contained the OE resistance mutant H274Y (designations of resistance mutations as recommended by Ferraris and Lina [9]), considered to be of relevance for OE resistance in human subjects [9]. In addition, altogether 7,342 N2 sequences of human (n = 5,866) or avian (n = 1,476) sources were analyzed, of which six human sources and one avian source genes contained the R292K mutation, considered to be of relevance for OE resistance in human subjects. The sequences analyzed were derived from strains collected between year 1933 and 2012 (more than 90% later than 2000), and sequence data were obtained from the GISAID (Global Initiative on Sharing Avian Influenza Data) EpiFlu™ Database. The search parameters were as follows for respective type A influenza virus: Host: Human and Avian, Location: all, Full genome: yes, Required Segments: HA and NA. Detailed information about all strains harboring any of the above mentioned resistance mutations are listed in Additional file 1: Table S1, Additional file 2: Table S2, Additional file 3: Table S3, Additional file 4: Table S4, Additional file 5: Table S5, Additional file 6: Table S6.

### Phylogenetic and recombination analysis

The un-rooted phylogenetic trees were constructed using the dnadist and neighbor joining programs included in the phylip package [13], using default settings. The search for homologous recombination was carried out by using the phi-test [14], and the methods RDP, GENECONV, Bootscan, MaxChi, Chimaera, SiScan, 3Seq, and LARD included in the RDP package [15].

## Results

**Figure 1:**
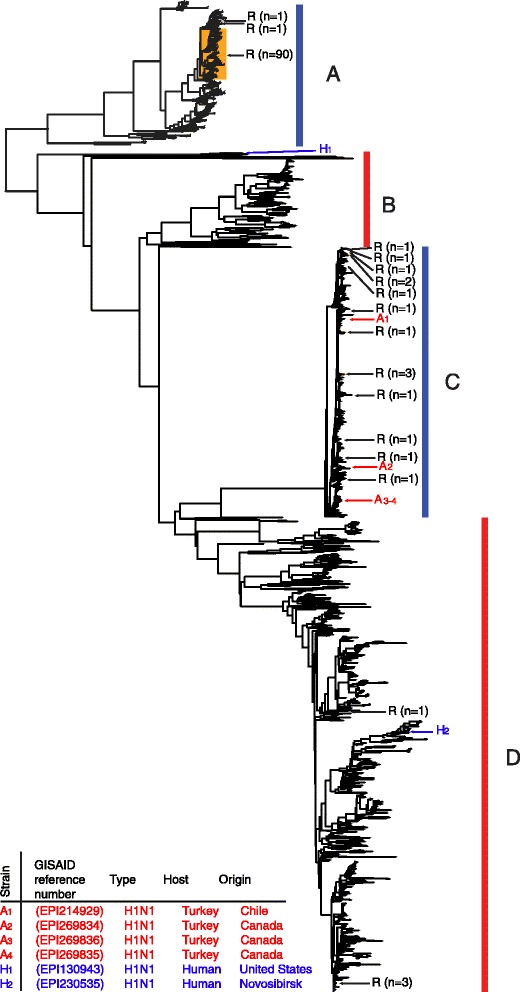
**Phylogenetic analysis of 616 N1 genes derived from H1N1 virus collected from humans and 1,009 N1 genes from influenza virus (irrespective of hemagglutinin identity) in avian samples.** The sequences represent a randomly chosen subset of the total set of altogether 12,413 N1 sequences analyzed. All 111 sequences containing the OE resistance mutation H274Y are included in the dendrogram. N1 sequences of virus collected from birds, are indicated by red vertical bars, and N1 sequences, derived from human samples, are indicated by blue vertical bars. N1 sequences from bird samples that map in clusters of sequences from human samples are denoted by red arrows, whereas sequences from human samples that map in clusters of sequences from avian samples are denoted by blue arrows. Details for these sequences are given in the bottom right section of the figure. Sequences carrying an OE resistance mutation are indicated by black arrows and, when clustered, also by a dark yellow box. Different clusters of sequences are designated **A-D** in block capitals.

A phylogenetic analysis was performed based on all sequences from influenza virus N1 genes from human and avian sources. Due to the large number of strains, the dataset was divided into nine subsets prior to the analysis. The dendrogram shown in Figure 1 is based on a randomly chosen sub-fraction consisting of 111 N1 sequences containing the H274Y resistance mutation together with sequences from 616 N1 genes of human source and 1,009 N1 genes of avian source. As expected, the sequences segregated into separate clusters, where Cluster A represented seasonal influenza viruses circulating before the A(H1N1)pdm09, Cluster C the pandemic A(H1N1)pdm09 influenza N1 sequences, and B and D represented N1 sequences of viruses collected from animals, mostly birds. Analysis of the other eight subsets of sequences presented similar divergences into these clusters. Hereafter, sequences clustering in A and C are referred to as human N1 sequences, whereas sequences clustering in B and D are considered as avian sequences. Cluster A contained 92, and cluster C contained 15, N1 sequences of human source with the H274Y OE resistance mutation whereas only four N1 sequences containing this mutation were detected in cluster D and none in cluster B (avian sources). Among all 10,351 N1 genes sequences from H1N1 strains collected from humans only two, one American (Figure 1, designated H1 in Cluster B) and one Siberian (H2 in Cluster D), appeared among the avian sequences in the phylogenetic tree. Four specimens from domestic birds but none from wild birds contained typical human N1 sequences, clustering with sequences from the pandemic H1N1 (Cluster C). All N1 genes sequences with the H274Y resistance mutation that were derived from human specimens were found among the typical human N1 sequences in Clusters A and C, clearly distinct from the two avian N1 genes of Cluster D.

**Figure 2:**
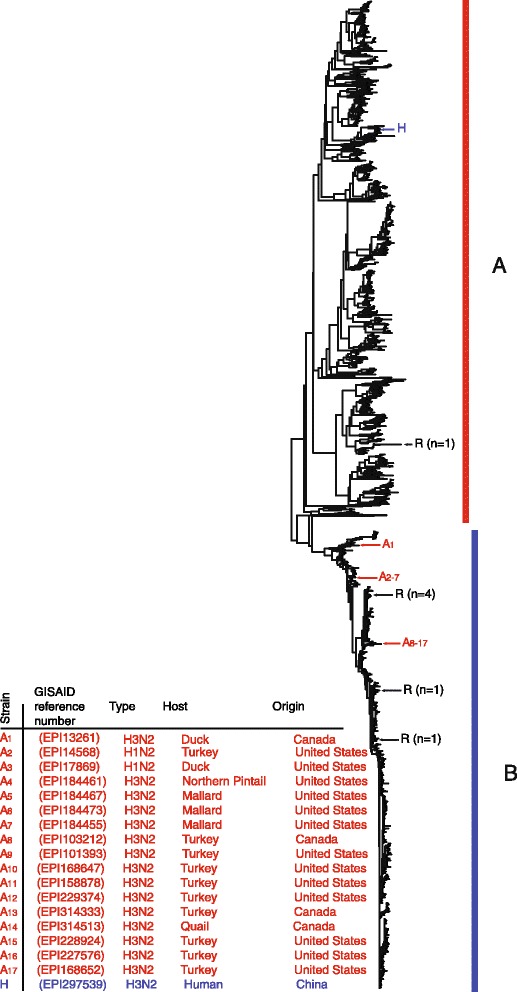
**Phylogenetic analysis of N2 genes derived from 1,254 H3N2-positive samples taken from humans and N2 genes from 1,450 N2-positive (irrespective of hemagglutinin identity) avian samples.** The sequences represent a randomly chosen subset of the total set of altogether 7,342 N2 sequences analyzed. All seven sequences containing the OE resistance mutation R292K are included in the dendrogram. N2 sequences derived from birds are indicated by red vertical bars, and N2 sequences from human samples are indicated by blue vertical bars. N2 sequences from bird samples that map in clusters of sequences from human samples are denoted by red arrows, whereas sequences from human samples in clusters of sequences from avian samples are denoted by blue arrows. Details for these sequences are given in the bottom right section of the figure. Sequences carrying an OE resistance mutation are indicated by black arrows. The two major clusters of sequences are indicated by block capitals **A** and **B**.

A corresponding analysis was performed for N2 sequences of human (n = 5,866) or avian (n = 1,476) sources. The oseltamivir resistance mutation R292K was identified in six N2 genes of human and in one of avian source. As with N1, the complete dataset of N2 strains was divided into subsets prior to phylogenetic analysis. The phylogenetic tree (Figure 2) includes all genes containing the R292K resistance mutation (n = 7) together with a randomly chosen sub-fraction (n = 1,247) of the sequences from humans and all the N2 genes of avian origin. The sequences segregated in two clusters, one representing human (Cluster B) and one representing avian N2 sequences (Cluster A). Analysis of the other subsets presented similar divergences into these two clusters. One N2 sequence from a human source was found among the N2 sequences from avian in cluster A (Figure 2), whereas altogether 17 N2 sequences from avian were found among the N2 sequences with human source (cluster B). In conclusion, among the 5,866 N2 sequences from H3N2 viruses identified in human samples, only one contained an N2 gene that grouped with the avian sequences, i.e. indicated an avian origin. Thus, transfer of avian N1 or N2 genes (with or without the H274Y or R292K resistance mutations) to H1N1 and H3N2 viruses circulating in the human population appear to be very rare.

In addition to H274Y or R292K, we also analyzed the whole set of 12,413 N1 and 7,342 N2 sequences for the presence of other resistance mutations (I117V, E119V, D198N, I222V, N294S and I314V). In total, any of these additional resistance mutations were identified in 137 N1 sequences (48 avian, 89 human) and 103 N2 sequences (31 avian, 72 human), but in none of these did we observe signs of transfer of resistance between human and avian strains by reassortment or recombination, as shown in Additional file 7: Figure S1 and Additional file 8: Figure S2.

The recent demonstration of intra-segmental homologous recombination in influenza A virus [16] has raised the question whether the OE resistance mutations could have been transferred from avian N genes to human N genes via intragenic recombination between human and avian N genes. This would result in chimeric N genes in H1N1 or H3N2 virus with avian as well as human sequence elements. We searched for homologous recombination between avian and human N1 or N2 genes using the phi-test and methods included in the RDP package, i.e., the methods RDP, GENECONV, Bootscan, MaxChi, Chimaera, SiScan, 3Seq, and LARD. The analysis was performed on all large datasets included in this study. No sign of intra-segmental homologous recombination between human and avian N1 or N2 genes was observed by any of the applied methods (p = 0.937 according to the phi test). To avoid inference of multiple testing owing to the large number of similar strains without resistance mutations, we also performed all tests on smaller datasets in parallel. These datasets contained only the consensus strains of all major clades (marked as A to D in Figure 1A and B in Figure 2), and the strains containing the resistance markers. Nor in these datasets could we find any sign of intra-segmental homologous recombination between human and avian N1 or N2 genes resulting in transfer of resistance mutations.

## Discussion

The present study, based on analysis of more than 19,700 complete N1 and N2 sequences in specimens from humans infected with H1N1 or H3N2 virus, shows that transfer of avian N genes to human influenza virus via re-assortment or intra-segmental homologous recombination is a rare event, irrespective if the avian N genes carry resistance mutations or not. A range of OE resistance mutations in avian influenza virus have been reported, depending on the geographical location of the bird populations [6,17], but their frequency in influenza viruses circulating in wild birds is overall low [17]. Interestingly, the H274Y resistance mutation was the only one found by Järhult and coworkers in mallards under experimental conditions with environmental OC concentrations [7], indicating that OC resistance patterns are similar in avian and human influenza H1N1 viruses. However, the present data indicate that the vast majority of H274Y and R292K resistance mutations evolve in the human influenza N1 and N2 genes without involving re-assortment, the major mechanism for exchange of genetic material between influenza viruses [18], with corresponding avian influenza genes. The low propensity of re-assortment between avian and human N1 viruses observed here is in line with the data by Obenauer et al. [19]. The absence of evidence for homologous recombination between human and avian influenza N1 and N2 genes is in line with previous results that homologous recombination, frequently occurring in DNA viruses [20], after all is a rare phenomenon in the evolution of influenza and other negative strand viruses, although a few exceptions have been described [21-25].

It cannot be excluded that transfer of resistance mutations from avian influenza N genes ever occur, or that resistance mutations may be transferred and then suppressed below the detection limit due to reduced fitness. However, whereas in case of chronic HIV or hepatitis B virus infection such minor mutant strains may persist even after termination of antiviral treatment, this is unlikely for influenza virus which does not cause chronic infections in humans. Instead, OE antiviral treatment of humans has proven sufficient to induce essentially all OE resistance that has been detected in human influenza until today by selection of mutations within N1 and N2 genes in strains that circulate in humans. Accordingly, the threat to human health from OE resistance emerging as result of treatment of infected humans appears to be much greater than the risk posed by transfer of OC resistance induced in avian influenza virus. This conclusion is not surprising considering the stronger selection pressure for enrichment of resistance mutations in oseltamivir-treated patients with serum OE concentrations of up to 500 μg/L [26], compared with more moderate concentrations (up to 30 μg/L) that avian influenza viruses encounter in animals close to sewage outlets from waste-water purification plants or adjacent watercourses [4,27,28].

A limitation of the present study is that it includes only avian and human sequences. Thus, we cannot conclude anything about the potential transfer of N genes or resistance mutations between birds and swine. Another limitation is that we have not analyzed OE resistance mutations in non-human influenza virus types such as H5N1, H6N1, H9N2, or H7N2, obtained from humans or birds. Thus, we have not assessed if humans have been infected with avian strains of these types that carry OE resistance induced by environmental exposure. However, these types are dead ends because they cannot be transmitted further to other humans, and the absence of avian clustering among the 17,693 N1 or N2 sequences of human source indicate that transfer of OE resistance by this mechanism is not important. Furthermore, due to the large number of strains, no bootstrapping was included in the phylogenetic analysis. Bootstrapping (bootscan) was, however, included in the recombination analysis performed using the RDP program.

Finally, it is important to stress that pollution of large amounts of oseltamivir or its active metabolic derivatives either as a consequence of manufacture or shedding from treated patients is unsatisfactory from another point of view. The status of OE as a stable, biologically non-degradable compound with the inherent capacity to be enriched in sensitive habitats raises concerns that OE may cause future but as yet unforeseen damage to wildlife in sensitive ecosystems [8].

## Conclusions

Our results presented here demonstrate that transfer of OE resistance mutations from avian to human N genes is extremely rare. It is therefore unlikely that resistance in influenza viruses infecting humans has been transferred from avian avian influenza A virus strains carrying mutations induced by environmental exposure to OC. Instead, resistance in influenza viruses infecting humans is most likely due to the selection pressure posed by the global OE administration in humans.

### Consent

No patients or persons were included in this study, and accordingly no consents were obtained.

## Additional files

Additional file 1: Table S1. Information about N1 strains harboring the H274Y, R292K resistance mutations. Additional file 2: Table S2. Information about N2 strains harboring the H274Y, R292K resistance mutations. Additional file 3: Table S3. Information about Human N1 strains harboring any of the I117V, E119V, D198N, I222V, N294S or I314V resistance mutations. Additional file 4: Table S4. Information about Avian N1 strains harboring any of the I117V, E119V, D198N, I222V, N294S or I314V resistance mutations. Additional file 5: Table S5. Information about human N2 strains harboring any of the I117V, E119V, D198N, I222V, N294S or I314V resistance mutations. Additional file 6: Table S6 Information about Avian N2 strains harboring any of the I117V, E119V, D198N, I222V, N294S or I314V resistance mutations. (XLS 15 kb) Additional file 7: Figure S1. Phylogenetic network of the N1 gene harboring strains with any of the resistance markers I117V, E119V, D198N, I222V, N294S and I314V. Non-resistant reference strains were included for comparison. Strains of Avian origin are highlighted in red and strains of human origin are highlighted in blue. Resistant strains have the type of resistance mutation as a prefix in the strain name. The network demonstrates no signs of reassortment or homologous recombination between avian and human strains. Additional file 8: Figure S2. Phylogenetic network of the N2 gene harboring strains with any of the resistance markers I117V, E119V, D198N, I222V, N294S and I314V. Non-resistant reference strains were included for comparison. Strains of Avian origin are highlighted in red and strains of human origin are highlighted in blue. Resistant strains have the type of resistance mutation as a prefix in the strain name. The network demonstrates no signs of reassortment between avian and human strains.
