# Supplementary figures and images for: Published sequences do not support transfer of oseltamivir resistance mutations from avian to human influenza A virus strains
Source: BMC Infect Dis. 2015 Mar 28;15:162. doi: 10.1186/s12879-015-0860-9 (PMC4387679; doi:10.1186/s12879-015-0860-9)

0.01

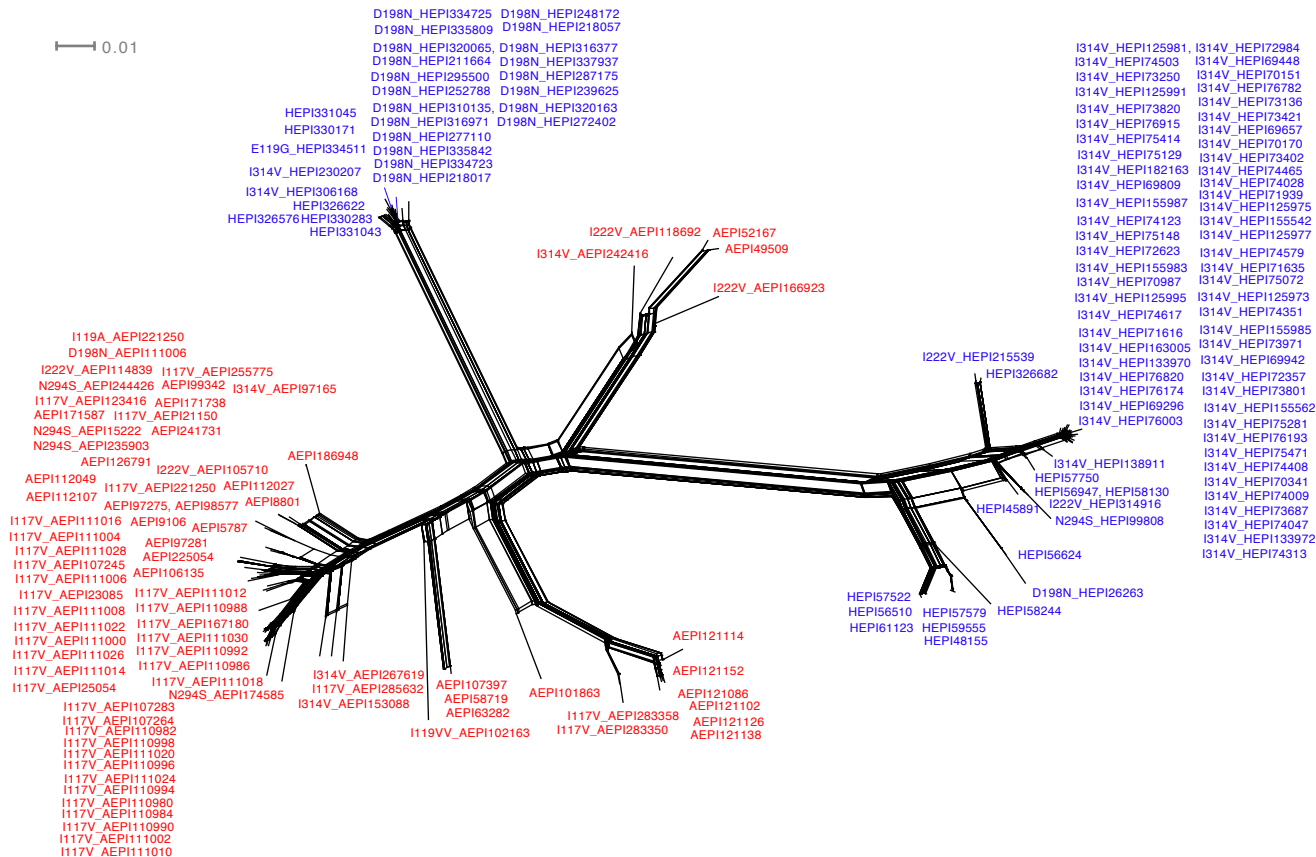

Supplement: Additional file 7: Figure S1. — Phylogenetic network of the N1 gene harboring strains with any of the resistance markers I117V, E119V, D198N, I222V, N294S and I314V. Non-resistant reference strains were included for comparison. Strains of Avian origin are highlighted in red and strains of human origin are highlighted in blue. Resistant strains have the type of resistance mutation as a prefix in the strain name. The network demonstrates no signs of reassortment or homologous recombination between avian and human strains. [file 12879_2015_860_MOESM7_ESM.pdf]

0.01

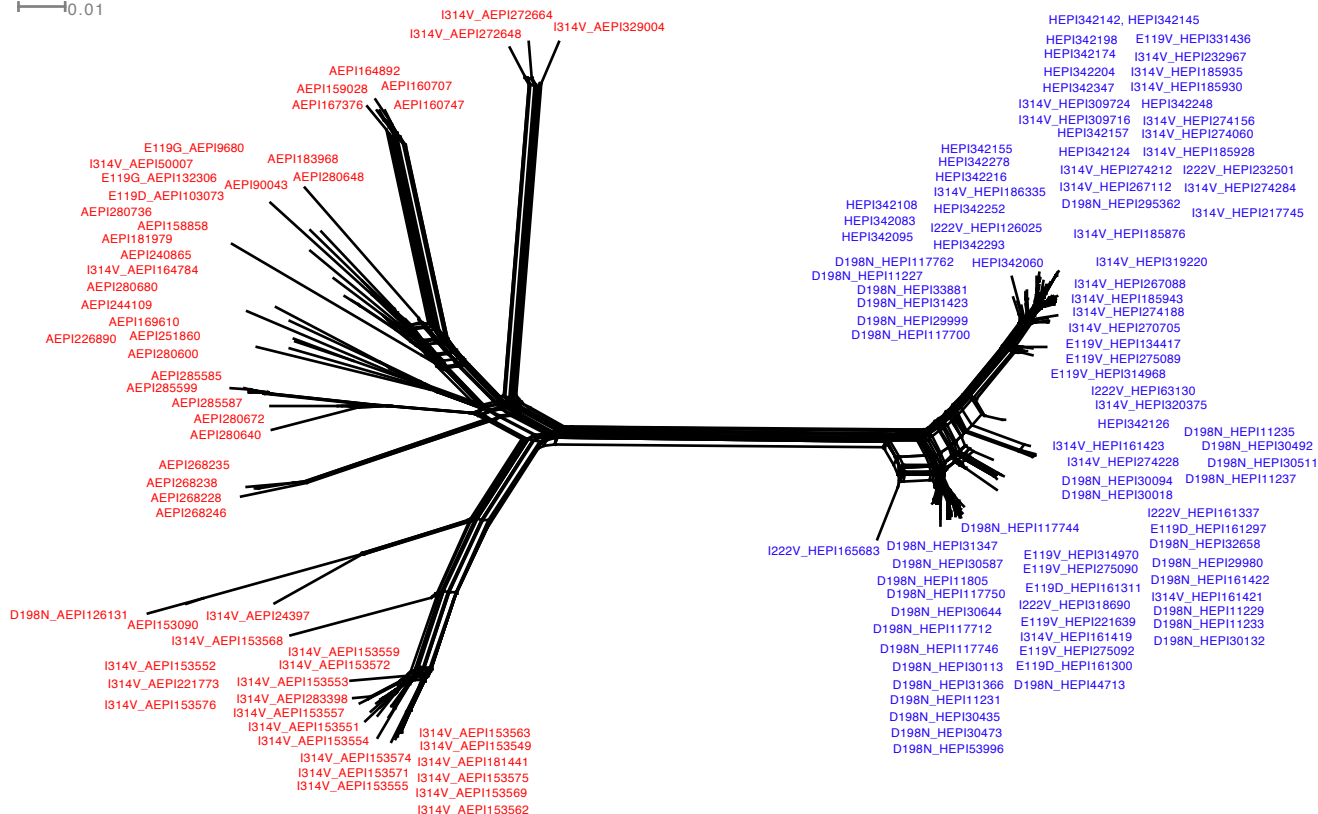

Supplement: Additional file 8: Figure S2. — Phylogenetic network of the N2 gene harboring strains with any of the resistance markers I117V, E119V, D198N, I222V, N294S and I314V. Non-resistant reference strains were included for comparison. Strains of Avian origin are highlighted in red and strains of human origin are highlighted in blue. Resistant strains have the type of resistance mutation as a prefix in the strain name. The network demonstrates no signs of reassortment between avian and human strains. [file 12879_2015_860_MOESM8_ESM.pdf]
